# Assessment of the Pulp/Tooth Ratio and Third Molar Root Completion Status for ‎Forensic Age Estimation in an Iraqi Population

**DOI:** 10.7759/cureus.89236

**Published:** 2025-08-02

**Authors:** Salam N Jawad, Safa H Alwan

**Affiliations:** 1 College of Dentistry, Bilad Alrafidain University, Baqubah, IRQ; 2 College of Dentistry, Al-Farahidi University‎, Baghdad, IRQ

**Keywords:** forensic age estimation, iraqi population, panoramic radiography, pulp/tooth ratio, third molar

## Abstract

Objectives: This retrospective cross-sectional study evaluates the effectiveness of using the pulp/tooth area ratio of mandibular second molars for identifying minors (<18 years) in an Iraqi population and compares its diagnostic performance to the third molar root completion status.

Methods: A total of 216 panoramic radiographs were analyzed. Pulp/tooth area ratios were measured using ImageJ (National Institutes of Health, Bethesda, MD), and third molar root completion was recorded as a binary variable. Three logistic regression models were constructed and evaluated using sensitivity, specificity, and area under the ROC curve (AUC).

Results: Incomplete third molar root development showed high sensitivity (0.90), while the second molar pulp/tooth ratio demonstrated superior specificity (0.99). The combined model (ratio + third molar) yielded the highest overall performance (AUC = 0.96), outperforming individual predictors (third molar only: AUC = 0.90; ratio only: AUC = 0.84).

Conclusion: While the pulp/tooth ratio alone is less sensitive, it provides excellent specificity and enhances overall model performance when combined with third molar status. These findings support the combined use of both variables for accurate age estimation in forensic settings lacking reliable documentation.

## Introduction

Age estimation is a critical component of forensic practice, particularly in determining legal responsibility [1]. In Iraq, individuals under 18 years of age are subject to different legal standards than adults, making reliable age estimation methods essential. This need is especially pressing in regions affected by conflict, where official documentation may be incomplete or lost [2].

Dental radiographs are commonly employed in age estimation due to the predictable patterns of dental development and aging [3]. After tooth development is complete, age-related regressive changes, particularly the deposition of secondary dentin, result in a progressive reduction of the pulp chamber. Several studies have used this phenomenon to estimate chronological age by measuring pulp dimensions relative to the tooth [4,5].

Cameriere et al. developed a method that combines third molar development with the pulp/tooth area ratio of the mandibular second molar to estimate whether individuals are above or below 18 years of age [5]. While validated in Italian and other European populations, the applicability of this method to Middle Eastern populations remains underexplored.

This study aimed to evaluate the diagnostic performance of the second molar pulp/tooth ratio in identifying minors within an Iraqi sample and to compare it with the more commonly used third molar completion status.

## Materials and methods

Sample selection

The study sample included 273 digital panoramic dental tomographs from patients attending the College of Dentistry, Al-Farahidi University (Baghdad, Iraq) for routine dental treatment. Ethical approval was obtained from the university's local ethical committee (ref. no. D.Eth./4, May 21, 2025). Inclusion criteria required a clear representation of the second molar without restorations or pathology. After excluding poor-quality radiographs, 216 cases remained (97 men and 119 women), with ages ranging from 12 to 65 years. Among them, 105 were minors (<18 years) and 111 were adults.

Radiographic analysis

**Figure 1:**
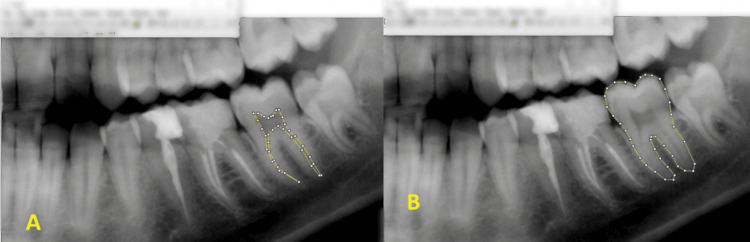
Selection of pulp (A) and tooth (B) outlines with polygon selection tool using ImageJ

Panoramic images were captured using the Gendex® GXDO-300 Orthopantomogram system (Gendex Dental Systems, Hatfield, PA) and analyzed with ImageJ® (National Institutes of Health, Bethesda, MD). The overall tooth and pulp outlines of the mandibular left second molar were traced using the polygon selection tool (Figures 1A, 1B). The pulp/tooth area ratio (R) was calculated based on pixel values. Third molar root development was recorded using Demirjian and Goldstein's classification [6], where status H was considered complete (Tm = 1), and all other stages were considered incomplete (Tm = 0). Sex was also included as a nominal variable.

Reliability assessment

To evaluate measurement reliability, a second observer repeated measurements on 30 randomly selected images before formal data collection. Interobserver agreement was assessed using Cohen's kappa.

Statistical analysis

Three binary logistic regression models were constructed using minor status (Mn = 1 for minors, Mn = 0 for adults) as the dependent variable. Independent variables included the pulp/tooth ratio (R), third molar status (Tm), and sex. Model performance was evaluated using sensitivity, specificity, and overall accuracy at a 0.5 probability threshold. Receiver operating characteristic (ROC) curves were plotted for each model, and area under the curve (AUC) values were calculated. Statistical analysis was conducted using Statistical Package for the Social Sciences version 23.0 (IBM Corp., Armonk, NY), with significance set at p < 0.05.

## Results

Interobserver reliability was strong for both the pulp/tooth area ratio (R) and third molar root completion status (Tm), with Cohen’s kappa values of 0.862 and 1.000, respectively. Three logistic regression models were constructed to predict minor status, as summarized in Table 1.

**Table 1:** Summary of logistic regression models used for predicting minor status R: pulp/tooth area ratio; Tm: third molar root completion status

Model	Predictors included	Description
(a)	R, Tm, sex	Full model including all candidate predictors
(b)	R, Tm	Sex excluded due to nonsignificance; best overall performance
(c)	R only	Simplified model using only R

Table 2 summarizes the classification performance of the three models, including sensitivity, specificity, AUC, and confusion matrix components. All performance metrics were calculated at a threshold of 0.5.

**Table 2:** Sensitivity, specificity, and AUC values for logistic regression models in minor status ‎classification ^*^Statistical significance was set at p < 0.001 Data are presented as point estimates with 95% confidence intervals. Sensitivity and specificity were calculated at a threshold of 0.5 Tm: third molar root completion (Tm = 1: complete, Tm = 0: incomplete); R: pulp/tooth area ratio; Sensitivity: correct identification of minors; Specificity: correct exclusion of adults; CI: confidence interval; TP: true positive; FN: false negative; TN: true negative; FP: false positive; AUC: area under the curve

Model (n = 216)	Sensitivity (95% CI)	Specificity (95% CI)	TP	FN	TN	FP	AUC	Overall accuracy	Chi-square
Model (a): Tm only	0.90 (0.85-0.96)	0.92 (0.85-0.96)	93	12	102	9	0.90^*^	91.76%	159.24^*^
Model (b): R + Tm	0.70 (0.60-0.78)	0.95 (0.89-0.98)	73	32	105	6	0.96^*^	89.81%	145.67^*^
Model (c): R only	0.54 (0.44-0.64)	0.99 (0.95-1.00)	57	48	110	1	0.84^*^	90.74%	124.83^*^

**Figure 2:**
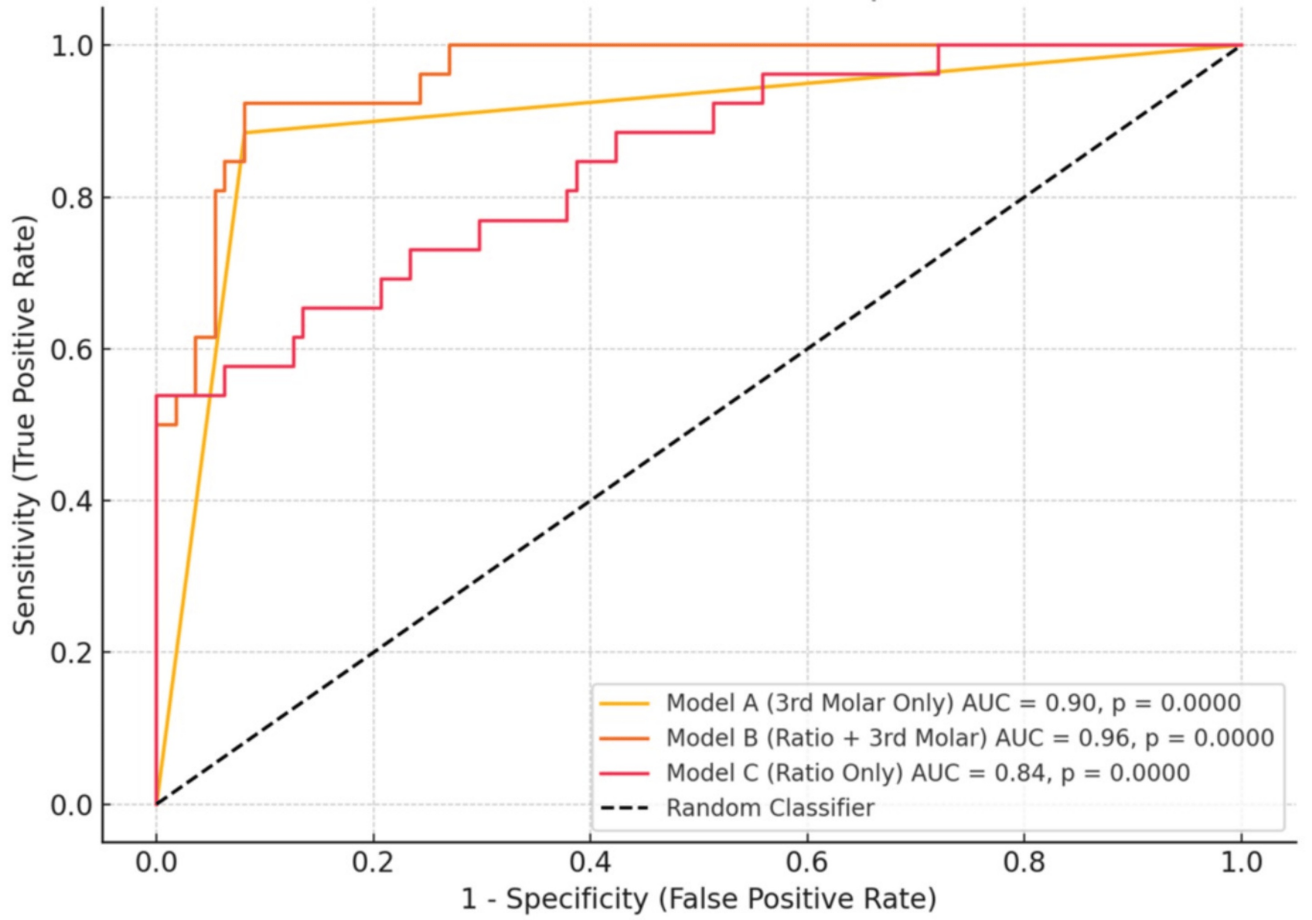
ROC curves for three predictive models of minor status ROC: receiver operating characteristic; AUC: area under the curve

ROC curves for the three models are shown in Figure 2. All models demonstrated statistically significant discrimination (AUC > 0.80, p < 0.001), with Model (b) achieving the highest AUC (0.96), reflecting excellent overall predictive performance.

Model (b), which incorporated both R and Tm, showed the highest AUC of 0.96, reflecting the best overall discriminatory performance across thresholds. These findings indicate that while third molar root completion status offers the highest sensitivity, the pulp/tooth ratio adds diagnostic value through superior specificity. This complementarity is visually confirmed in the ROC analysis (Figure 2), which shows the combined model (b) achieving the greatest overall discriminative power.

## Discussion

Accurate age estimation is essential for protecting minors' legal rights and ensuring appropriate application of the law [7]. This is particularly relevant in Iraq, where decades of conflict have led to the loss or unreliability of official records [2]. In such contexts, dental radiographs offer an objective and accessible means of determining legal age, which is particularly important to asylum proceedings, undocumented refugee assessments, or child protection investigations, where accurate and noninvasive age estimation is critical [8,9].

Third molar root development has long been a cornerstone of forensic age estimation [1,7], due to its variability and late maturation. However, its sensitivity often comes at the expense of specificity. Conversely, the pulp/tooth ratio of second molars, affected by secondary dentin deposition, offers a more conservative indicator of age, potentially reducing the risk of false-positive classification of adults as minors [3-5].

Our findings corroborate previous research by demonstrating high sensitivity (0.90) for third molar root completion in detecting minors [1,7]. However, the pulp/tooth ratio model achieved outstanding specificity (0.99), making it valuable in minimizing false-positive classifications of adults. When combined in logistic regression models, these variables yielded high overall classification accuracy.

In addition to the classical metrics mentioned earlier, the discriminative performance of each model was confirmed through ROC analysis. The combined model (pulp/tooth ratio and third molar status) yielded the highest AUC of 0.96 (p < 0.001), indicating excellent predictive accuracy across a range of thresholds. Even the third molar-only model achieved a strong AUC (0.90), while the pulp/tooth ratio model alone showed good discrimination (AUC = 0.84), though limited by its low sensitivity.

While the pulp/tooth ratio alone is insufficient for identifying all minors due to its lower sensitivity (0.54), it significantly enhances specificity when used alongside third molar status. This suggests a complementary role for the pulp/tooth ratio in forensic contexts, particularly when avoiding misclassification of adults as minors is paramount.

These findings are consistent with several studies underlining the limitations of depending solely on third molar development, particularly in diverse ethnic groups [10-12]. Local validation is required because the pulp/tooth ratio has shown population-specific variance in its diagnostic accuracy [13,14]. When combined with established measures, readily available techniques like panoramic radiography offer a dependable yet practical alternative in resource-constrained or conflict-affected environments like Iraq. Finally, more comprehensive evaluations of dental age estimation techniques, such as Demirjian and Goldstein [6] and other morphological protocols, highlight the trade-offs between specificity and sensitivity based on the technique and the population under study [15].

A limitation of this study is the use of year-only birth data, which precluded precise age calculations. Moreover, the sample's single-institution origin may limit the generalizability of findings to other ethnic or regional populations. Further studies with exact birthdates and larger, more diverse Iraqi subpopulations would be valuable to enhance validity.

## Conclusions

The pulp/tooth area ratio of mandibular second molars, while less sensitive, offers excellent specificity and significantly enhances the discriminative performance of age estimation models when combined with third molar root completion. The high AUC values observed confirm the combined model's robustness, supporting its use as a reliable forensic tool in contexts where official documentation is unreliable or unavailable.
